# Evaluation of functional differences between two anticoagulation methods used in continuous renal replacement therapy in critical patients

**DOI:** 10.1186/cc13585

**Published:** 2014-03-17

**Authors:** V Goulão, E Lafuente, J Silva, M Fernandes, F Santos, A Ferreira

**Affiliations:** 1 CHTS, Penafiel, Portugal

## Introduction

The aim of this study is to analyze the functional alterations that may influence the final result when using citrate versus heparin anticoagulation in critically ill patients [1].

## Methods

We performed a retrospective and analytical study including patients exclusively submitted to citrate or heparin through years 2011 and 2012. Included were demographic data with SAPS II, SOFA, RIFLE scores and mortality rate. For functional analysis we consider the timing of the beginning, duration, loss of dose and loss of creatinine clearance. We analyzed dialitrauma, considering the variation of: potassium, total and ionized calcium, magnesium, sodium, phosphorus, pH, lactates, bicarbonate, platelets, albumin, creatinine and urea. Data are presented as the average and standard deviations. To access the influence of dose and clearance losses on mortality, we used logistic regression test.

## Results

The study included 44 patients in the citrate group versus 61 in the heparin group. We found no statistical significant differences for: age (*P *= 0.06); SAPS II (*P *= 0.28); SOFA (*P *= 0.19); the timing of beginning of the technique (*P *= 0.61), with 34.8% versus 47.5% of patients in R (RIFLE), 27.8% versus 18% in I (RIFLE) and 16% versus 21% in F (RIFLE); duration of the technique (*P *= 0.74) and length of stay. Although we noticed a greater loss of dose and absolute creatinine clearance in the citrate group, this had no statistical significance (*P *= 0.18 and *P *= 0.13). The mortality found for citrate and heparin groups was 60.4% and 39.4% respectively. The differences with statistical significance related to dialitrauma emerged in K+ (*P *= 0.03), Ca^2+ ^(*P *= 0.02), Na+ (*P *= 0.004), platelets (*P *= 0.002), pH (*P *= 0.02) and bicarbonate (*P *= 0.0001). Logistic regression for mortality in the citrate versus heparin groups showed the following values: effective dose (ROC 0.435 vs. ROC 0.663), clearance (ROC 0.606 vs. ROC 0.663), SAPS II (ROC 0.482 vs. ROC 0.696), SOFA (ROC 0.713 vs. ROC 0.696) and RIFLE (ROC 0.695 vs. ROC 0.636).

## Conclusion

We may say that there are functional differences that must be taken into account. Despite not having statistical significance on this sample, losses of dose and creatinine clearance showed a direct relation with mortality.
